# Vision-Based Efficient Robotic Manipulation with a Dual-Streaming Compact Convolutional Transformer

**DOI:** 10.3390/s23010515

**Published:** 2023-01-03

**Authors:** Hao Guo, Meichao Song, Zhen Ding, Chunzhi Yi, Feng Jiang

**Affiliations:** 1 School of Computer Science and Technology, Harbin Institute of Technology, Harbin 150001, China; 2 School of Medicine and Health, Harbin Institute of Technology, Harbin 150001, China

**Keywords:** bio-inspired design and control of robots, robotics, reinforcement learning, vision transformer

## Abstract

Learning from visual observation for efficient robotic manipulation is a hitherto significant challenge in Reinforcement Learning (RL). Although the collocation of RL policies and convolution neural network (CNN) visual encoder achieves high efficiency and success rate, the method general performance for multi-tasks is still limited to the efficacy of the encoder. Meanwhile, the increasing cost of the encoder optimization for general performance could debilitate the efficiency advantage of the original policy. Building on the attention mechanism, we design a robotic manipulation method that significantly improves the policy general performance among multitasks with the lite Transformer based visual encoder, unsupervised learning, and data augmentation. The encoder of our method could achieve the performance of the original Transformer with much less data, ensuring efficiency in the training process and intensifying the general multi-task performances. Furthermore, we experimentally demonstrate that the master view outperforms the other alternative third-person views in the general robotic manipulation tasks when combining the third-person and egocentric views to assimilate global and local visual information. After extensively experimenting with the tasks from the OpenAI Gym Fetch environment, especially in the Push task, our method succeeds in 92% versus baselines that of 65%, 78% for the CNN encoder, 81% for the ViT encoder, and with fewer training steps.

## 1. Introduction

Complex autonomous controlling in robotic manipulation tasks has been possible, profiting from the development of the Reinforcement Learning (RL) method. Notable successes about RL built on various video games [1,2,3,4], continuous control tasks such as learning legged locomotion [5,6], and manipulation skills which are represented by robot grasping [7,8] and in-hand object re-orientation [9] in simulation environments and the real world. Furthermore, Vision-based RL, which utilizes high dimensional observations, generates flexible control policies and increases the possibility of the general application of RL in the real world. The agent in the vision-based RL algorithm interacts with the environment based on the raw sensory observation instead of assuming other artificially designed access to the state information [10,11,12,13,14].

However, current methods of learning from visual observation in RL mainly focus on solving the data efficiency issue, but to some extent ignore the issue of policy robustness. Previous investigations used Sim2Real [7,15,16], imitation learning [17,18,19,20], data augmentation [21,22], and other methods to overcome the barrier of data efficiency in real or simulated robot application, preliminarily attain satisfying accomplishments. Nevertheless, the general performance of policy needs to be improved as well. Among the basic robotic manipulation tasks, previous studies tend to get unbalanced results in each task, and it is difficult to decrease the prominent gaps in performance. For visual-based RL, the quality of input visual features directly affects the training process and final performance. The policy stability, which consists of the performance fluctuation in multi-tasks and the same task in diverse environments, is dominated by the features extracted from visual observation. Meanwhile, general performance for different tasks is precisely related to object isolation in various visual environments. Hence, in this paper, we argue that, besides the efficient policy structure, an efficient image encoder, which serves as the visual features extractor, that focuses on extracting the valid position features of objects and operators, weakens the distracting visual information in the surrounding environment for policy optimization, which is another fundamental requirement to guarantee a robust system that operates the feedback visual observation.

As for the visual feature extractor, current methods often utilize the CNN, to some extent, the collocation of RL policies and ConvNets indeed achieves high efficiency and success rate on some tasks [21,22,23,24]. However, the performance of a currently used CNN feature extractor relies on the numerous training data, and the data efficiency advantage of the original framework could be debilitated due to the exclusively increased cost of the encoder optimization

In this paper, we propose DCCT: Dual-streaming Compact Convolutional Transformer, an effective RL framework with a Transformer-based visual encoder that significantly improves policy general performance for vision-based robotic manipulation with dual-camera views, taking advantage of the superiority of the lite ViT [25] and preserving the RL method efficiency. Specifically, each view is encoded individually using the single channel of the dual-streaming visual encoder in DCCT (The details of DCCT hyperparameters are listed in (Appendix A)), features extracted from the ConvNet in encoder comprise spatial information naturally, casting off the extra positional embeddings in original ViT. Features from each channel are fused before entering the linear layer. Features fusion compensates for the spatial information demand of single view and unifies the visual attention in different views. After encoder procession, the visual features are utilized as the input of framework to optimize the policy end-to-end until convergence.

To maintain the extant advantage of data efficiency, we employ contrastive unsupervised learning, data augmentation in an offline policy RL, as the foundational framework of our method [24]—first, collecting and storing ten demonstrations in a replay buffer; second, initializing the dual-streaming encoder based on demonstration data with unsupervised contrastive pre-training; third, training the offline policy RL framework based on the augmented data from demonstrations and images collected during online training.

Recent work has certified that a dual-camera combination of egocentric and third-person views, which offers the compound of the complementary interesting area data in local and global vision fields, is more robust than the setup of a single monocular camera wherever the lens is fixed [7,24]. This meets the physiological principle found by Land et al. that the oculomotor system will focalize the center of sight close to the point where the visual target is extracted when humans perform a complex task. Third-person views contain master view and over-the-shoulder (OTS) view, and each of the views has been used as the third-person view option. However, to the best of our knowledge, there is no study on the choice of third-person views in the combination of egocentric and third-person views. In this paper, we study the effectiveness of the alternative third-person views with the combination observations, demonstrating the advantage of master view versus the OTS view based on the attention explainability of ViT in three general robotic manipulation tasks.

To verify the effectiveness of our method, we extensively evaluate our method in the OpenAI Gym Fetch environment and compare it to the ConvNet-based state-of-the-art (SOTA) methods: FERM [24] and SVEA [8] with three tasks: Reach, Push, and Pick-and-Place. Relying on the effectiveness of DCCT, we maintain data efficiency and improve the performance of the ConvNet-based method. Especially in the Reach task, our method notably improves final performance and diminishes the discrepancy among each task, achieving a success rate of 92% with the fewest training steps, versus 65%, 78%, and 81% for FERM, SVEA (CNN encoder), and SVEA (ViT encoder), respectively. Finally, in the article we present, we highlight the main contributions below: (i) We explore the DCCT, which significantly improves the method general performance in three tasks widely used for generalization, as a novel efficient robotic manipulation framework based on RL; (ii) Based on the dual-streaming visual encoder and the observations of the master view and the egocentric view, we raise a dual-streaming process architecture to achieve the data fusion and leverage the dual-view information to improve manipulation precision. (iii) According to the attention explainability of ViT, in scenarios of the robot manipulation: reaching, picking and placing, and pushing an object. We experimentally observe that the master view outperforms the OTS view, on final performance and efficiency in the combination with the egocentric view.

## 2. Related Work

### 2.1. Data-Efficient Transformer

Since the introduction of attention mechanisms from language processing to computer vision, ViT and their variants have shown extraordinary results in vision tasks. However, the “data-hungry” peculiarity still impedes the generation of data-efficient requirements. The trend of research on lite Transformer is burgeoning [26,27,28,29]. Touvron et al. [30] proposed Data-Efficient Image Transformer (DeiT) which applies a novel knowledge distillation procedure to reduce the data dependence of ViT and perform well on mid-sized datasets such as ImageNet and fine-tune small datasets such as CIFAR-10, both for image classification. Hassani et al. [25] raised three progressive ViT models: ViT-Lite, Compact Vision Transformer, and Compact Convolutional Transformer to break the dependence of large sets of data, with suitable smaller patch sizing, sequential pooling method, and convolutional based patching method, respectively. While these results are emboldening that the Transformer can perform better accuracy with SOTA CNNs on small data sets. Our work also leverages the method architecture of [25] as the basic visual module and illuminates the general performance and data efficiency of our proposed encoder based on the Transformer.

### 2.2. Visual Learning for RL

Extensive research has been investigated to improve the data efficiency of vision-based RL [31,32,33]. Recent developments in vision-based RL have heightened the need for data augmentation and unsupervised representation learning. Zhan et al. [24] and Li et al. [34] achieve extremely sample-efficient training of robotic manipulation SAC and offline RL via TD3+BC policies respectively that utilize data augmentation and unsupervised learning. However, the oppression of data efficiency compels the regular CNN visual encoder to extract object features unsteadily. In consideration of the outstanding performance of the Transformer, many works have studied the combination of Transformer and RL [35,36,37,38,39]. This type of combination made some improvements in partial visual-based tasks, but the preponderance of ViT is not steady and even worse than the original visual encoder which may be due to the massive data requirements in general tasks training. Getting rid of the “data-hungry” peculiarity and preserving the excellent performance of ViT are two of the challenges to be overcome. In this work, we achieve data efficiency and general performance improvement with the DCCT visual encoder, unsupervised contrastive learning, and data augmentation. At the same time, we also compare the SOTA method with original ViT in Section 4.2. Therefore, our method has the potential application to a larger variety of robotic manipulation tasks.

### 2.3. Contrastive Learning

Contrastive learning, as a kind of self-supervision method, learns the general representations that obey the unsupervised similarity constraints in a dataset combined with similar and dissimilar point pairs, with unlabeled data. Relative to the focus on pixel details of generative learning, the latent capacity to concentrate on abstract information is learned in the contrastive learning model by minimizing the distances between similar data clusters and maximizing the distances between dissimilar data clusters. Contrastive learning has notable progress in the past couple of years with huge advances in vision [40,41,42]. To date, Chen et al. [42] and Laskin et al. [21] have investigated the data augmentation for contrastive learning and performed the preponderance of the representations that were pre-trained with transferred data. In this work, we leverage the instance-based contrastive learning and pre-training that have been verified in computer vision and RL in simulated environments.

### 2.4. Data Augmentation

Data augmentation [43,44,45] improves the general performance of model with a limited number of data, under the condition of an unsubstantial increase in data. Inspired by the previous work, the most relevant to our work is visual augmentation. Laskin et al. [22] show that data augmentations alone can significantly improve the data efficiency and generalization of RL methods operating from pixels, without any changes to the underlying RL algorithm. Hansen et al. [8] show that data augmentation in deep Q-learning algorithms drastically improves model stability and generalization. A considerable amount of studies have substantiated that random crops stably improve sample efficiency while most data augmentation methods increase sample efficiency but also increase divergence risk. In this work, we leverage random crop visual augmentation in our robotic manipulation framework to improve generation and data efficiency.

## 3. Method

**Figure 1:**
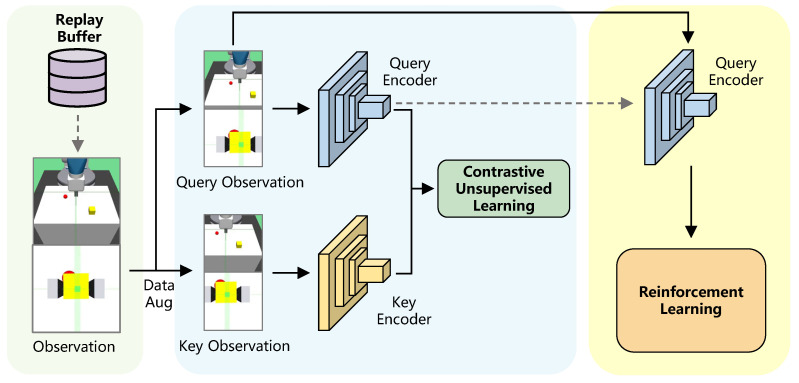
Architectural overview. A pair of observations are sampled from the replay buffer, and observations are then transformed into query and key observations by data augmentation. The data-augmented observations are encoded with the query and key encoders, respectively, for pretraining the encoder with contrastive unsupervised learning. The query encoder with initialized parameters is continually updated to train the agent in RL algorithm with data augmentation.

For the sake of data efficiency in agent training, we utilize a framework that contains three modules: demonstration buffer, contrastive unsupervised learning with dual-streaming encoder, and reinforcement learning with data augmentation to achieve an efficient RL policy. The architecture overview of our framework is depicted in Figure 1, and the details are introduced as follows.

### 3.1. Demonstration Buffer

According to the results from Zhan et al. [24], ten-demonstration is the proper setting that can satisfy the balance point between efficiency and training performance, while our work also uses demonstrations for unsupervised contrastive pre-training. In response to this situation, we set the buffer with ten demonstrations in each simulated Gym environment, including Fetch Pick-and-Place, Fetch Reach, and Fetch Push.

### 3.2. Contrastive Unsupervised Learning with Visual Encoder

After the initialization of the replay buffer, we train the dual-streaming encoder for the input image with contrastive learning. Contrastive learning, one of the categories of representation learning, can be explained as a dictionary look-up task that is differentiable.

The query–key pair in contrastive learning generated relies on random crop data augmentation. Query and key, which can be also called anchor and positive, are the data pair that needs to maximize the agreement, while positive and negative are the data pair that needs to minimize the agreement. Two different augmentations of the same image were set as the anchor and positive observation separately while other images were set as the negatives. According to the relationship among the three parts, given a query *q* and keys $K=\{k_{0},k_{1},\ldots \}$, then *K* is segmented into $k_{*}$ and $K/k_{*}$, the intention of this design is to ensure the *q* matches with $k_{*}$ better than any other $k_{i}$ in $K/k_{*}$, $q,k_{*},K,K/k_{*}$ can also be known as anchor, positive, target, and negative. There are many methods to measure the match performance of *q* and *k*, such as dot products $q^{T}k$ or bilinear products $q^{T}Wk$ while other forms such as Euclidean distances are similarly common.

Considering the characteristic of unsupervised learning, there is no label in datasets or any other prior knowledge that can be the evidence to extract negatives directly. Consequently, contrastive learning needs to set a loss function that can maximize the score of $\left(q,k_{+}\right)$ pair and minimize the $\left(q,k_{-}\right)$ pair, learning the most significant features in similarity relations relative to the negative pairs. With the joint optimization of Noise Contrastive Estimation (NCE) and mutual information, the infoNCE, which is proposed by [46], can be interpreted as a multi-class cross entropy classification loss of a K-class softmax classifier, and meet requirements of the unsupervised contrastive learning. The infoNCE loss is described as follows:

(1) $$L_{q}=\log\frac{\exp\left(q^{T}Wk_{+}\right)}{\exp\left(q^{T}Wk_{+}\right)+\sum_{i=0}^{K-1}\exp\left(q^{T}Wk_{i}\right)}$$

According to the results in FERM, for simulated environments and some simple tasks in the real world, the pretraining of the image encoder, which is constructed by a stack of CNN networks, fails to bring a remarkable benefit in task performance. At the same time, with the appearance of Transformer as the SOTA models in NLP and CV, the combination of CV and Transformer justified the ViT has the potential to surpass the traditional CNN models in CV. However, without the inductive biases inherent to CNNs, the Transformer lost the abilities such as translation equivariance and locality, which led to a belief that the performance of Transformer relies on the model size and the amount of training data. Considering the data hungry characteristic of ViT, which sets a barrier for data-efficient robot training, we train a model based on the work of [25], which is suitable for small sets of data and retains a SOTA performance for image encoding to ensure the pre-training phase’s performance and data efficiency.

As far as we know, DCCT is the first method to introduce the lite ViT concept in robotic manipulation framework. To better use the lite ViT, which was designed for classification in small data sets originally, we eliminate the SeqPool part which is used to map the sequential outputs to a singular class index in the original lite ViT. At the same time, different from fusing data of each channel primarily in the previous double views process [7,24], we set the data fusion part at the latter of visual encoder, to extract the individual features from each channel as more as possible, keeping more complementary information from two views, since the narrow dimensionality reduction or data filtration for the single channel will influence the confidence proportion from each view, and it can avoid data redundancy based on its structure setting.

#### 3.2.1. ViT Encoder Architecture Overview

**Figure 2:**
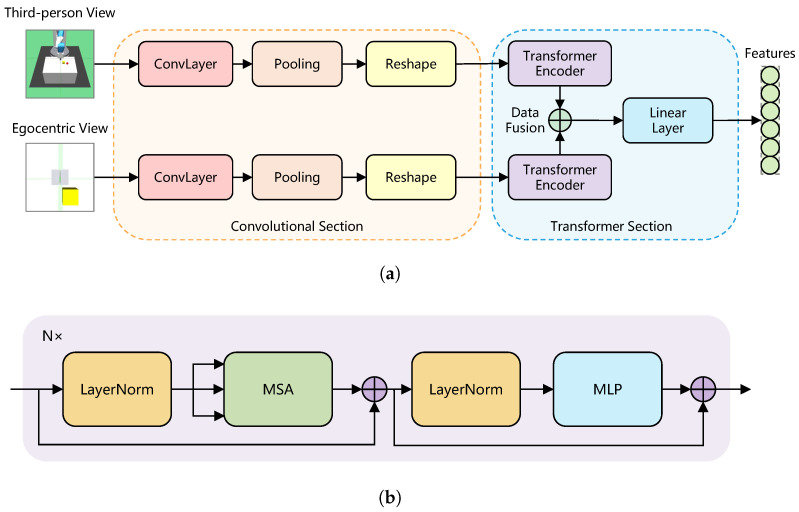
ViT encoder architecture. Observations from each view are divided into respective process channels. The images are encoded to patches and be tokenized by pooling and reshaping for the Transformer, and the output vectors of each Transformer encoder are data fused into a single vector, which is extracted features by the final linear layer. (**a**) DCCT visual encoder; (**b**) Transformer encoder.

The lite ViT model we proposed contains two sections: the convolutional section and the Transformer section. For original ViT, the input image is divided into many patches and realigned into a sequence, which is the suitable input format for Transformer; at the same time, positional embedding is an indispensable part which can provide spatial information for the sequence data. According to [25], for small datasets, a smaller size patch is better than the original size patch, and the patch size matters for the performance of ViT. In order to satisfy the basic conditions and guarantee efficient data training, we introduce a convolutional section into the image encoder, or in other words, we introduce the attention mechanism into the CNN encoder mechanism. The padding method based on the convolutional section can provide local information and spatial information about the relationships between patches together, without the additional learnable or designed positional embeddings. The details of the image encoder are shown in Figure 2.

#### 3.2.2. Convolutional Section

From the view of the Transformer model, for the sake of preserving the inductive bias character of the original image coder, the convolutional section is designed to replace the functions of images patching, positional embedding, and image embedding. The convolutional section adopts the traditional convolutional combination mode, which contains two convolution layers with ReLU activation, and a max pool layer, the input image $i\in R^{C\times H\times W}$, the output o:

(2) $$i_{0}=MaxPool\left(ReLU\left(Conv2d\left(i\right)\right)\right)$$

To match the embedding dimension of the Transformer backbone, the number of the filters in conv2d is the same as the embedding dimension. At the end of the convolutional section, the max pooled data are reshaped in a sequence, which is proper to enter the Transformer section. The overlapping of the convolutional kernels stride increases the information density which is easily neglected when it appears at the boundary of the patches in original ViT, since the significant image information may be broken and lost its original value after it is divided into two or more parts, even the addition of the positional embeddings cannot reveal the initial affection. In addition to this, overlapping can raise the performance to rely on the inductive bias. Compared to ViT, the utilization of the convolutional section divides the image into patches in a more flexible way, and the image is embedded into a potential representation that is more suitable and efficient for the Transformer encoder.

#### 3.2.3. Transformer Section

The Transformer section consists of a Transformer encoder and a linear layer, referencing the structure of the original ViT, and the Transformer encoder is constituted with a Multi-headed Self-Attention (MSA) layer and a Multi-Layer Perceptron (MLP) head. Layer normalization applied in the Transformer encoder for data normalization, activation function applied GELU, and applied residual connection to prevent the network degeneration. After the Transformer encoder, the output data from each channel are data fused into a single tensor, dimension reduction of tensor is processed with linear layer, and generating the features that are suitable for the RL algorithm.

### 3.3. Reinforcement Learning with Data Augmentation

With sufficient pretraining, the DCCT visual encoder outputs the treated data to the RL phase. Among the RL algorithms, in support of the stability and the potentiality, the on-policy algorithm recently became popular. However, there exists a restriction that an on-policy algorithm demands several millions even billions of action executions in the environment, which is often daunting for real robot training. Other than the on-policy algorithm, the off-policy algorithm is a natural fit for robot training from pixel information in the real world, since it is independent from the hypothesis that samples are coming from the current trained policy, which means it can reuse the samples multiple times without over-fitting in complex visual tasks.

Soft Actor Critic (SAC), an off-policy method, was justified its effectiveness in learning to walk on a real quadruped robot [6] with just 2 h from scratch, and learning manipulation skills in simulated gym environments [21,22,23]. According to this, we set SAC as an agent to interact with the environment with augmentation. Taking count of the augmentation categories in simulated environments and the efficiency and practicality in real robots training, we extract a random patch from the original frame as the augmentation image in contrastive pre-training. During updating gradient, the agent receives a mixture of demonstration data and training collected data.

#### Soft Actor Critic

SAC is a SOTA off-policy algorithm that optimizes a stochastic policy for maximizing the expected trajectory for continuous control. With the utilization of data augmentation, SAC extends its advantage of efficiency from state observation to pixel observation [21,22,24], even achieving the same extent of state observation in simulated benchmarks. SAC, an actor–critic method, learns a policy $\pi_{\phi }$ and critics $Q_{\phi_{1}}$ and $Q_{\phi_{2}}$. The critic parameters $\phi_{i}$ are learned by minimizing the squared Bellman error:

(3) $$L\left(\phi_{i},B\right)=E_{t∼B}\left[{\left(Q_{\phi_{i}}\left(o,a\right)-\left(r+\gamma \left(1-d\right)T\right)\right)}^{2}\right]$$

where $t=(o,a,o^{\prime },r,d)$ is the tuple with observation *o*, action *a*, reward *r*, and done signal *d*, B is the replay buffer where the demonstration observations are stored, and T is the target that the critics are trained to match, defined as:

(4) $$T=\min_{i=1,2}Q_{\phi_{i}}^{*}\left(o^{\prime },a^{\prime }\right)-\alpha \log\pi_{\psi }\left(a^{\prime }|o^{\prime }\right)$$

where $Q_{\phi_{i}}^{*}$ is the exponential moving average (EMA) of the parameter of $Q_{\phi_{i}}$. The application of EMA can improve training stability in off-policy RL algorithms according to empirical research. α is a positive entropy coefficient that affects the priority of the entropy maximization over the optimizing target matching optimization.

In order to learn the desired actor policy, the actor samples stochastically from the policy $\pi_{\psi }$ and is trained to maximize the expected return as in:

(5) $$L\left(\psi \right)=E_{a∼\pi }\left[Q^{\pi }\left(o,a\right)-\alpha \log\pi_{\psi }\left(a|o\right)\right]$$

where $a_{\psi }\left(o,\xi \right)∼\tanh\left(\mu_{\psi }\left(o\right)+\sigma_{\psi }\left(o\right)⊙\xi \right)$, ξ∼N(0,I) is a standard normalized noise vector.

## 4. Experiments

In this section, we investigate the avail of the attention mechanism in the pixel-based efficient robotic manipulation training framework via processing image observations in the DCCT visual encoder before sending them as the agent in the RL environment. We evaluate the performance and efficiency maintaining of our method with the baseline of FERM, SVEA (CNN encoder), and SVEA (ViT encoder). At the same time, we separately set the ablation experiments to investigate the contribution of each section in our method.

### 4.1. Experiments Setup

We evaluate our proposed framework in the OpenAI Gym robotics environment, which is based on the simulated goal-based tasks for Fetch robots, including:

- Pick-and-Place: picking up a box from a table using its gripper and moving it to a desired goal above the table;
- Push: moving a box by pushing it until it reaches the desired goal position;
- Reach: moving its end-effector to the desired goal position.

**Figure 3:**
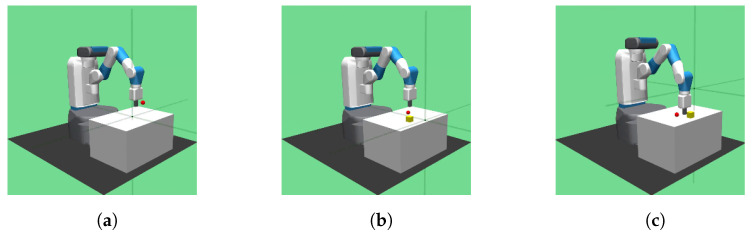
Experimental environments. Agents are trained in OpenAI Gym Fetch simulated environment, including three robotic manipulation tasks: Reach, Pick-and-Place, and Push. (**a**) Reach; (**b**) Pick-and-Place; (**c**) Push.

Compared to the setup of the tasks in FERM, the Reach, Pickup, and Move tasks in FERM can respond to the Reach, Pick-and-Place, and Push tasks in the simulated environment, respectively. Among the FERM tasks, Pickup and Move tasks, in spite of obtaining a good performance at the final evaluation, cost a significantly long optimization time relative to the other tasks, especially the Move tasks, which cost the longest time but the lowest success rate. The three tasks in the OpenAI Gym Fetch environment are shown in Figure 3. At the same time, we apply a combination of a master view and an egocentric view as the dual-camera views and a further discussion is described in Section 4.3.4

As mentioned in FERM, for simulation and easier tasks in their suite, the unsupervised pre-training had no significant benefit in performance, to investigate and improve the unsupervised pre-training performance, we adopt the set of pre-training in each task. In the meantime, we set another exciting investigation from Hansen, et al. [8] as a reference baseline work. They proposed a general RL framework which achieves stabilized Q-value estimation under data augmentation, leveraging two data streams. At the same time, SVEA investigated both CNN and ViT as the visual encoder; this work achieves both high data efficiency and performance and also attains the SOTA performance.

### 4.2. Results

In this section, we mainly compare the results which reveal the performance and data efficiency from our investigation. To weaken the affection of CPU or GPU calculated power, we use the interaction steps number rather than interaction time to evaluate the data efficiency, and the final successful rate to evaluate the final training performance. The interaction steps include the steps for policy optimization, which means the steps cost to convergence, and the steps to 90% success rate, which represents that the method has reached a high-performance level. Results for comparison are shown in Table 1. Below are the key findings:

1. The four items in Table 1 show that the DCCT method we proposed significantly improves the performance and training data efficiency in the three simulated robotic manipulation environments compared to the FERM, SVEA (CNN encoder), and SVEA (ViT encoder). Especially for the Push task, our method reaches the highest final success rate and the sole method reached the 90% success rate. As for the other tasks, besides the full mark success rate as the other methods, our method also achieves the least steps to the convergence, which means the cost of the policy optimization time of our method is the least;
2. Comparing the steps difference value of 90% success rate and convergence, our method costs the least steps in each task, from a high-performance stage to the stabilization stage;
3. Based on the steps statistics and the average time of each step, the DCCT costs slightly more training time than FERM in Reach and Pick-and-Place tasks; meanwhile, apparent time efficiency relative to SVEA (CNN encoder) and SVEA (ViT encoder) appears. Especially for the Push task, DCCT reveals an obvious time efficiency advantage in comparison with the other methods. With the requirement of efficiency, the preponderance of lite ViT versus original in robotic manipulation can also be revealed in this comparison.
4. As we can see from Table 1, the Push task is the most difficult task which costs the maximum train steps and obtains the lowest final success rate. However, what leads to this situation? In our opinion, the other tasks can be treated as the one-stop mode tasks that during the period of end-effector reaches the target district or leaves to the next point, there is nothing else that limits the trajectory planning, the physical parameter will not affect the end-effector to replan trajectory. However, for push, the shape of the block and end-effector, the contact position, the moving direction of the end-effector, and other physical parameters all lead the block to deviate from its original trajectory together. Policy of Push needs to refresh trajectory much more frequently in single task term than other tasks, which is extremely adverse in the policy training period.

### 4.3. Ablations

Based on the efficiency certification of demonstrations and data augmentation in FERM, we focus on three aspects of our method: the tokenization method for the Transformer section, the Transformer encoder, and unsupervised pretraining, to investigate the affection of image encoder based on lite ViT to the training framework. At the same time, we also investigate the alternatives in the third-person views to confirm the optimum view combination with egocentric view.

#### 4.3.1. Patching Method for Transformer

**Figure 4:**
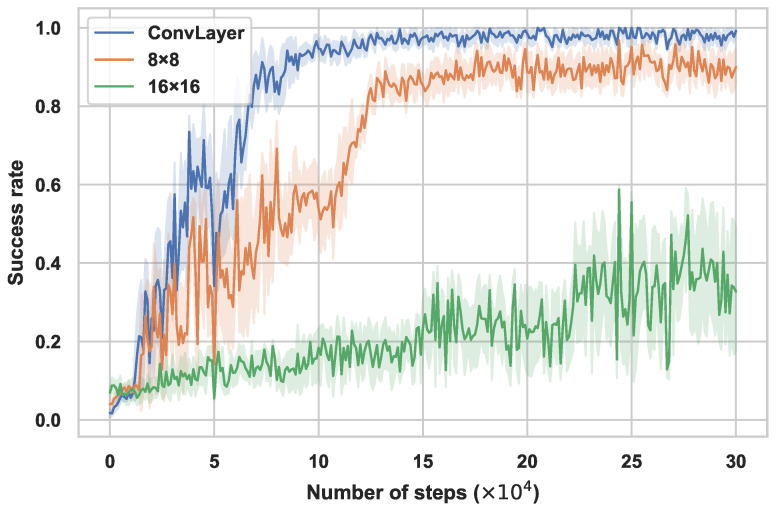
Performance of patching method ablation. Training performances (success rate) of ConvLayer, 8 × 8 and 16 × 16 patching methods in Push task. Mean and std. deviation of 25 seeds.

To investigate the impact of the image patching method for the Transformer section, we evaluate our method by comparing embedding the image into patches with CNN and ordinary image segmentation. For image segmentation, besides the original 16 × 16 patch size in ViT, we also utilize 8 × 8, a smaller patch size that is more suitable for small datasets. After segmentation, we apply linear projection and reshaping for the embeddings into the encoding layer of the Transformer. Meanwhile, the image embeddings are added with positional embeddings to reserve spatial information before entering the Transformer section. The success rate results during the training phase are shown in Figure 4.

It is obvious that the method with the original ViT patch size 16 × 16 fails to converge and the small patch size 8 × 8 and the CNN encoder both converge to a high success rate, the barely satisfying results of the original patching size method may due to the lack of inductive bias and the character of data hungry, which can be rectified by increasing the number of images for pretraining. However, for the experience of the ViT, the data requirement of a qualified original ViT is several times of CNN, which is extremely disadvantageous in an efficient method. Furthermore, relative to the small patches method, the replacement of the CNN encoder raises the converge speed and performance level.

Based on the image patching method ablation results and FERM, which only applies the CNN as image encoder cannot attain an extraordinary performance in this task, to some extent, the comparison verifies the potent power of the combination of Transformer and the patching method based on CNN in the robotic manipulation training framework.

#### 4.3.2. Transformer Encoder

To investigate the impact of the Transformer section, we set the comparison experiments with the method retained Transformer versus the method without the Transformer in Reach task. In order to adapt the sequential token input format of the Transformer, we also set some format transition parts in our method. In the ablate comparison experiment, we use the single CNN part as the image coder to replace the Transformer.

**Figure 5:**
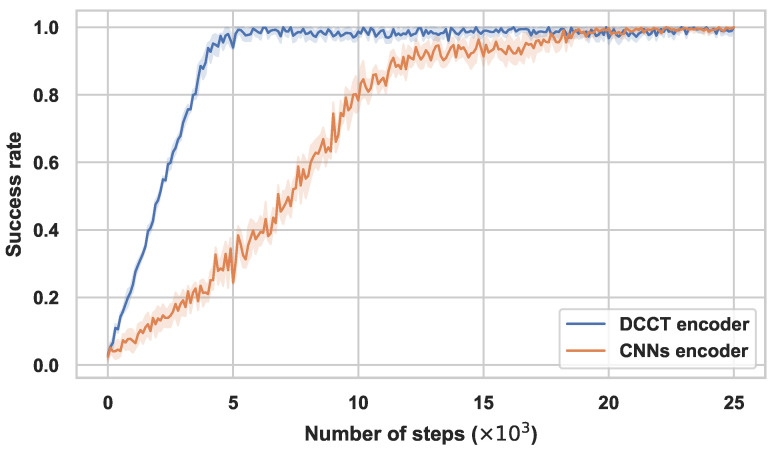
Performance of Transformer encoder ablation. Training performances (success rate) of with-Transformer encoder and without-Transformer encoder in Reach task. Mean and std. deviation of 25 seeds.

The comparison results are shown in Figure 5, referencing the results in Table 1, since the Reach task is a simple task; the method with a CNN encoder also converges to a high success rate, although the convergence speed of method with a CNN encoder is slightly reduced compared to the FERM, and the quantity of convergence steps in DCCT is nearly half of the method with the CNN encoder. Without the assistance of Transformer, the framework weakens the power of learning features in policy optimization. The disparities in convergence speeds and performances become enlarged when training difficult tasks according to Table 1.

#### 4.3.3. Unsupervised Pre-Training

**Figure 6:**
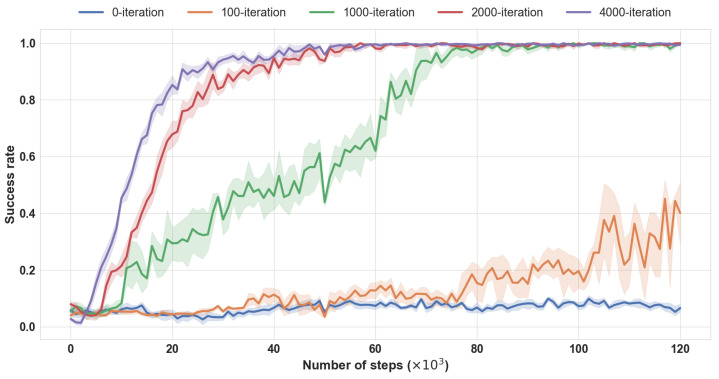
Performance of pre-training iterations ablation. Training performances (success rate) of with pre-training iteration times in Pick-and-Place task. Mean and std. deviation of 25 seeds.

To investigate the impact of the unsupervised pretraining based on Transformer in our method, we set five comparison experiments in Pick-and-Place task with 0, 100, 1000, 2000, and 4000 pre-training iterations in the encoder initialization, and the results are shown in Figure 6. We find that, with 0 or 100 iterations, the agent fails to learn an optimal policy. With the increment of pre-training iterations, the comprehensive performance of the agent is getting better, adequate pre-training is able to accelerate convergence in the policy optimization process and keep the optimal policy in a small fluctuation range. The advantage of 4000-iteration relative to 2000-iteration is not as prominent as the advantage of 2000-iteration to 1000-iteration, and 2000-iteration gratifies the demand for learning efficiency and performance.

#### 4.3.4. Third-Person View

**Figure 7:**
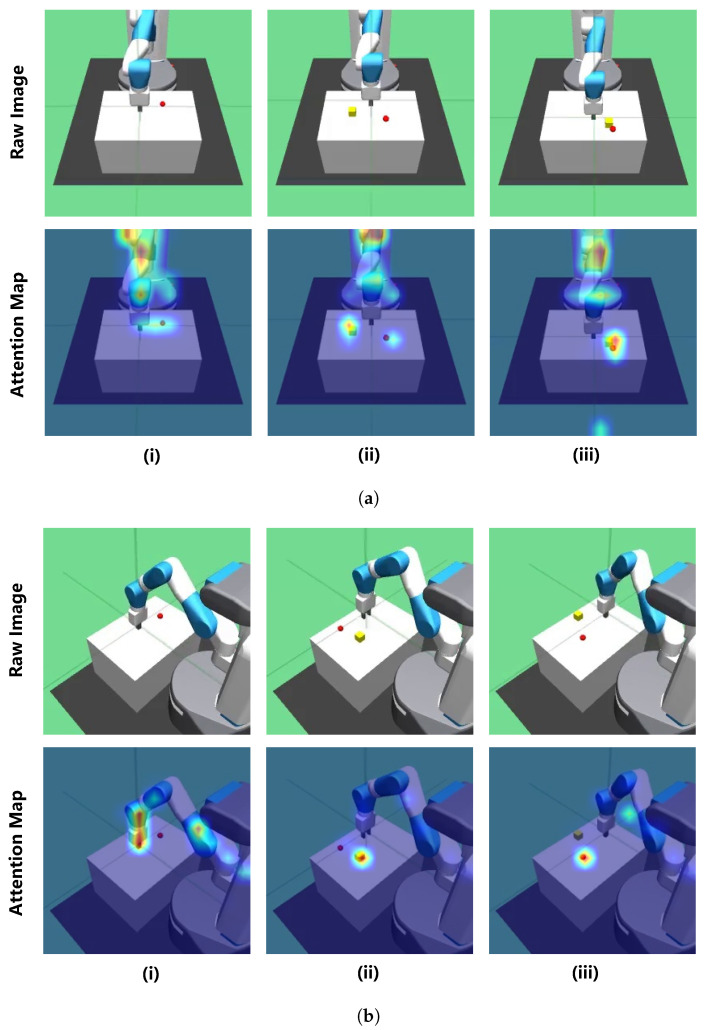
Attention maps. The visualization of attention maps from the Transformer encoder. For each subgraph, each column demonstrates task category: (i) Reach, (ii) Pick-and-Place, and (iii) Push, respectively. The upper row exhibits the raw image of a specific view, and the lower row exhibits the corresponding attention maps. (**a**) comparison of master views; (**b**) comparison of OTS views.

**Figure 8:**
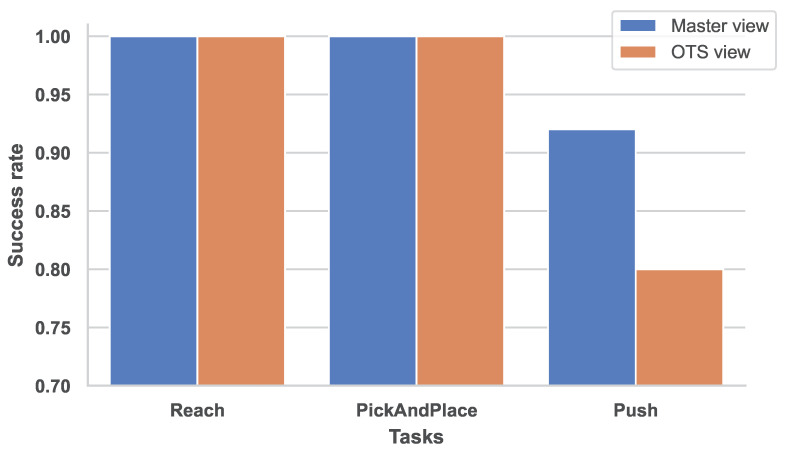
Performance of third-person view ablation. Policy performances (success rate) of dual-camera view with master view and OTS view in whole three tasks; statistical data of 25 seeds.

Although the application of dual-camera views improve the performance, to the best of our knowledge, there is no previous research investigating the third-person view mode ablation. To explore the effectiveness of the third-person view, we set an ablation experiment about the choice of the third-person view: OTS view and master view, which are depicted in Figure 7 with respective attention maps. For the OTS view, the visual feedback is fitter for the direct manipulation control than the master view. For the master view, the visual feedback has the best entopic observation of objects, but with inverse manipulation direction of the manipulator, which adds extra task complexity. The ablation results are shown in Figure 8, where we compare the success rates in each task.

Referencing the attention maps and corresponding results, it is obvious that the agent devotes itself to the regions of interest. For the master view, the interest regions cover the manipulator, object, and the target mark in each task. On the contrary, interest regions of the OTS view only focus on a single target, which may be distracted by the oversized manipulator arm occupied proportion in observation, and the changing outline of the moving manipulator arm. With the master view as the third-person view, a disparity in success rate has appeared, especially for the Push task. The master view reveals its superiority in the dual-camera setup and motivates us to explore more intricate tasks along with this mechanism.

## 5. Conclusions

The general performance of the vision-based efficient robotic manipulation in multi-tasks is a persistent dilemma. In this study, we have proposed DCCT, an effective RL framework with a novel vision encode method based on lite ViT that significantly improves the general performances of three robotic manipulation tasks in the OpenAI simulated environment. In our method, we propose the dual-streaming encoder to extract and integrate the visual information from double views synchronously, improving visual robustness among different tasks. At the same time, utilizing unsupervised learning, data augmentation, and SAC to achieve data efficiency in the training process. Furthermore, we experimentally demonstrate the dominance of the master view versus the OTS view in third-person views for our three tested tasks based on the attention visualization of ViT. Our study verifies the effectiveness of the lite ViT in an RL framework that allows for high effectiveness and with limited visual demonstrations. This research would be important for many application domains where multi-task general performance is required, but the training data are limited. Future work includes evaluating the general performance of our method in the real-world robot, extending the category of robotic manipulation tasks, and the reduction of the demonstration time.

## Figures and Tables

**Table 1:** Performance comparison in robotic manipulation. Task success rates of FERM, SVEA (CNN encoder), SVEA (ViT encoder), and Our method in the three simulated robotic manipulation environments, with ten expert demonstrations. Mean of 25 seeds.

Tasks	Metrics	Reach	Pick-and-Place	Push	Average Time of Each Step (ms)
	Steps to 90% success rate	8K	62K	-	
FERM	Steps to convergence	19K	78K	203K	32.2
	Final success rate (%)	100	100	65	
	Steps to 90% success rate	5K	51K	-	
SVEA (CNN encoder)	Steps to convergence	10K	72K	170K	102.3
	Final success rate (%)	100	100	78	
	Steps to 90% success rate	5K	64K	-	
SVEA (ViT encoder)	Steps to convergence	12K	83K	186K	128.6
	Final success rate (%)	100	100	81	
	Steps to 90% success rate	5K	45K	92K	
DCCT	Steps to convergence	9K	52K	109K	53.1
	Final success rate (%)	100	100	92	

## Data Availability

Not applicable.

## Abbreviations

The following abbreviations are used in this manuscript:

RL	Reinforcement Learning
ViT	Vision Transformer
DCCT	Dual-streaming Compact Convolutional Transformer
CNN	Convolutional Neural Network
SOTA	State-of-the-art
DeiT	Data-Efficient Image Transformer
FERM	Framework for Efficient Robotic Manipulation
MSA	Multi-headed Self-Attention
MLP	Multi-Layer Perceptron
SAC	Soft Actor Critic
SVEA	Stabilized Q-Value Estimation under Augmentation
OTS	Over-the-shoulder

## Appendix A

For each channel of the DCCT encoder, we utilize the same encoder architecture and hyperparameters. The details of DCCT hyperparameters are listed in Table A1.

**Table A1:** DCCT hyperparameters.

Hyperparameter	Value
Observation rendering	(100, 100)
Observation crop	(84, 84)
Hidden units (MLP)	1024
MSA	8
Optimizer	AdamW
$\left(\beta_{1},\beta_{2}\right)\to \left(f_{\theta },\pi_{\psi },Q_{\phi }\right)$	(0.9, 0.999)
$\left(\beta_{1},\beta_{2}\right)\to \left(\alpha \right)$	(0.5, 0.999)
Learning rate $\left(f_{\theta },\pi_{\psi },Q_{\phi }\right)$	2 $\times \;10^{-3}$
Learning rate (α)	2 $\times \;10^{-4}$
Weight decay	3 $\times \;10^{-2}$
Batch Size	512
Convolutional layers	2
Number of filters	36
Transformer encoder layers	6
Convolutional kernal size	3 × 3
Q function EMA T	0.01
Critic target update freq	2
Nonlinearity	ReLU
Encoder EMA T	0.05
Discount γ	0.99
Initial temperature	0.1
